# Neurodevelopmental Precursors and Consequences of Substance Use during Adolescence: Promises and Pitfalls of Longitudinal Neuroimaging Strategies

**DOI:** 10.3389/fnhum.2016.00296

**Published:** 2016-06-24

**Authors:** Diana H. Fishbein, Emma J. Rose, Valerie L. Darcey, Annabelle M. Belcher, John W. VanMeter

**Affiliations:** ^1^Bennett Pierce Prevention Research Center and The Department of Human Development and Family Studies, College of Health and Human Development, The Pennsylvania State UniversityUniversity Park, PA, USA; ^2^Center for Functional and Molecular Imaging (CFMI), Department of Neurology, Georgetown UniversityWashington, DC, USA; ^3^Department of Psychiatry, University of Maryland School of MedicineBaltimore, MD, USA

**Keywords:** longitudinal, prospective, substance use, adolescence, neuroimaging

## Abstract

Neurocognitive and emotional regulatory deficits in substance users are often attributed to misuse; however most studies do not include a substance-naïve baseline to justify that conclusion. The etiological literature suggests that pre-existing deficits may contribute to the onset and escalation of use that are then exacerbated by subsequent use. To address this, there is burgeoning interest in conducting prospective, longitudinal neuroimaging studies to isolate neurodevelopmental precursors and consequences of adolescent substance misuse, as reflected in recent initiatives such as the NIH-led Adolescent Brain Cognitive Development (ABCD) study and the National Consortium on Alcohol and Neurodevelopment (NCANDA). To distinguish neurodevelopmental precursors from the consequences of adolescent substance use specifically, prospective, longitudinal neuroimaging studies with substance-naïve pre-adolescents are needed. The exemplar described in this article—i.e., the ongoing Adolescent Development Study (ADS)—used a targeted recruitment strategy to bolster the numbers of pre-adolescent individuals who were at increased risk of substance use (i.e., “high-risk”) in a sample that was relatively small for longitudinal studies of similar phenomena, but historically large for neuroimaging (i.e., *N* = 135; 11–13 years of age). At baseline participants underwent MRI testing and a large complement of cognitive and behavioral assessments along with genetics, stress physiology and interviews. The study methods include repeating these measures at three time points (i.e., baseline/Wave 1, Wave 2 and Wave 3), 18 months apart. In this article, rather than outlining specific study outcomes, we describe the breadth of the numerous complexities and challenges involved in conducting this type of prospective, longitudinal neuroimaging study and “lessons learned” for subsequent efforts are discussed. While these types of large longitudinal neuroimaging studies present a number of logistical and scientific challenges, the wealth of information obtained about the precursors and consequences of adolescent substance use provides unique insights into the neurobiological bases for adolescent substance use that will lay the groundwork for targeted interventions.

## Introduction

A number of studies suggests that the adolescent brain may be particularly vulnerable to multiple adverse consequences of substance (i.e., alcohol or illicit drug) use (SU; e.g., Baker et al., 2013; Gulley and Juraska, 2013; Jacobus and Tapert, 2014; Petit et al., 2014; Spear and Swartzwelder, 2014; Spear, 2014, 2015; Squeglia et al., 2014; Smith et al., 2015) and subsequently, to development of SU Disorders (SUD; Guerrini et al., 2014). It is widely posited that protracted maturation of the prefrontal cortex (PFC) may be largely responsible for this period of heightened risk for SU, as adolescents have limited cognitive controls to engage in “top-down” behavioral regulation (Casey and Jones, 2010; Geier, 2013). Relative to limbic or subcortical structures instrumental in reward and emotion processing (e.g., ventral striatum and amygdala), neural substrates for behavioral, emotional, and cognitive regulation undergo a critical fine-tuning that increasingly connects its functions to those of limbic areas (Happaney et al., 2004; Casey et al., 2008; Casey and Jones, 2010; Blakemore and Robbins, 2012). This late-stage incubation and maturation of the neurophysiological architecture for “vertical control” in a variety of contexts ensures intact executive cognitive functioning (ECF) in adulthood (Brown and Tapert, 2004; Lenroot and Giedd, 2006). During adolescence, this frontal lobe development coincides with sequential improvement in ECF that supports behavioral self-regulation (Ahmed et al., 2015). In effect, the relative lack of vertical control in adolescence may be the single largest precipitant of adolescent SU.

Various neurocognitive deficits and abnormalities in brain structure, function and connectivity have been implicated in adolescents who misuse substances relative to alcohol and drug naïve subjects (e.g., Tapert et al., 2001, 2004; Nagel et al., 2005; Medina et al., 2007; Migliorini et al., 2013; Cousijn et al., 2014; Jacobus and Tapert, 2014; Whelan et al., 2014; Cuzen et al., 2015; Ramage et al., 2015; Weissman et al., 2015), suggesting that exposure to illicit substances itself may produce long-lasting neuronal alterations. Yet, most studies are cross-sectional and retrospective, and those that are longitudinal often do not include a true substance naïve baseline (e.g., Pfeifer et al., 2013; Sherman et al., 2014; Braams et al., 2015); thus, we have been unable to effectively disentangle neurodevelopmental precursors from frank neuronal insults that occur as a direct result of SU. Longitudinal studies with a substance naïve baseline are, therefore, necessary to elucidate variations in neuromaturation that influence emergent regulatory processes and in turn, predict onset and severity of eventual SU. It is also crucial to ascertain whether measurable delays in neurodevelopment are associated with the extent and escalation of SU, after controlling for “baseline” (i.e., prior to SU onset) neurocognitive functioning. This information will aid in the interpretation of observed neurocognitive deficits in substance users from studies largely focused on static, age-specific time points or those that include individuals who have initiated SU.

## Prospective, Longitudinal Designs to Isolate Consequences from Precursors of SU

Given the paucity of research delineating neurocognitive precursors and consequences of SU, particularly as they relate to the development of complex functions in adolescents, large-scale, tightly controlled panel studies (i.e., longitudinal studies with repeated measures from a single sample at multiple time points) are needed. These types of studies are uniquely able to examine two critical questions that can only be modeled through temporal sequencing. First, did some of the so-called “consequences”—neurocognitive deficits found in those who have initiated use—antedate and perhaps contribute to the onset and escalation of SU (i.e., are they liability factors)? This issue may be especially pertinent given that neurocognitive deficits have been implicated in propensity for SU and related problems (Mittenberg and Motta, 1993; Volkow et al., 2001; Simon et al., 2002; Ersche et al., 2006; Welch et al., 2013; Wilcox et al., 2014). Specific deficits in complex cognitive functions may act as risk factors for SU, and not simply consequences that deteriorate further with subsequent use. Several studies are beginning to flesh out the particular neurocognitive determinants of SU pathways, in interaction with psychosocial experiences and contexts (Fishbein et al., 2011, 2016a; Tarter et al., 2011; Kirisci et al., 2013b).

The second issue that can only effectively be addressed with a panel study approach is the impact of SU characteristics on neurodevelopmental trajectories. Adolescence is a particularly pertinent period for assessing the impact of SU on neurocognition, as demands for coping with competing social, cognitive, biological, and academic changes are high and have important long-term implications for developmental risk trajectories (McLaughlin et al., 2015). Based on early developmental vulnerabilities of neurocognitive functioning, it is plausible that the earlier, more frequent and intense SU is, the worse the outcome will be, and that neurodevelopmental processes evolving during this vulnerable period may be differentially impacted by light versus heavy SU. Thus, typologies of use, including indices of severity (i.e., frequency and quantity) at each time point must be considered. This consideration is even more pertinent in the context of prospective, longitudinal efforts, as youth who begin to use very shortly after they enter the study (regardless of age) and continue use will be exposed to substances for a longer period of time and may show greater cumulative effects. Thus, measures must assess level and rate of maturation of neurocognitive functions as they progress at varying stages of no SU, initiation, and types of continued use.

Despite a significant body of relevant developmental literature, there is limited information on how SU may stunt the progression of neurocognitive functions and cause a relative flattening in their developmental trajectories. A developmental perspective suggests that there should not be an expectation that level of functioning would necessarily decrease during adolescence; rather, it may either cease to develop, or develop more slowly or less fully. This approach has significant implications for interpreting neurocognitive assessment results, which have primarily involved individual or group comparisons between static age-related time points rather than extent of change over time. Efforts to delineate these effects are underway in a few longitudinal studies (Fishbein et al., 2016a).

## Prospective, Longitudinal Neuroimaging Designs: Value Added

Prospective, longitudinal studies that include neuroimaging offer distinct advantages over cross-sectional and non-prospective designs. Deficits in ECF and emotion regulation are underpinned by developmental patterns of brain activity and connectivity that vary by brain region and developmental time point. Elucidation of this differential development across relevant functions may provide more specific and sensitive information about risk factors for initiation and escalation of SU. The first advantage of neuroimaging techniques is that they allow for the characterization of structural and functional features at a substance-naïve baseline that may be predictive of SU and non-SU pathways (i.e., which individuals are more at risk to progress to SU and misuse versus those who will be comparatively resistant or resilient). Neuroimaging studies also promise to shed light on the “consequences” part of the equation by identifying adverse change in brain structure (e.g., volume) or function (e.g., neural activation and connectivity patterns) with subsequent use that may create a negative feedback loop with long-term consequences. Youth who subsequently misuse substances are expected to show protracted or attenuated neurodevelopment in key cortical structures, controlling for pre-use neurocognitive functioning and relevant covariates. Particular attention in these studies is being paid to neurodevelopment in brain regions that are implicated in behavioral profiles of interest (e.g., early SU onset and escalation, risky decision-making, emotion misattribution and dysregulation), such as orbital frontal (OFC), dorsal lateral prefrontal (dLPFC), medial prefrontal (mPFC), and rostral anterior cingulate cortices (rACC), striatum, amygdala, and insula, as well as emergent structural connectivity in cortico-limbic networks. SU-related effects in these areas may portend a critical exacerbation of pre-use deficits in ECF and emotion regulation and in turn, predict escalation to higher levels of SU and SUD. A longitudinal design using imaging also facilitates determination of whether more advanced levels of neurodevelopment before SU are a protective factor for those adolescents who don’t use or who use less frequently and in smaller quantities, or who potentially may incur less damage even with higher levels of use.

To-date, only a few prospective studies have been conducted using neuroimaging and an intensive battery of neurocognitive tasks sensitive to potential impact of early SU and eventual escalation on neurodevelopment throughout adolescence (Fishbein et al., 2016a). Intensive, empirically-guided neurocognitive and functional and anatomical neurologic exams, including specific task-based and resting state functional MRI (fMRI) measures and structural MRI techniques, such as diffusion tensor imaging (DTI), cortical thickness (CT), and voxel-based morphometry (VBM), are needed to fully characterize adolescent developmental maturation patterns and to identify mechanisms that are believed to be associated with, and affected by, SU. These neural activation and connectivity patterns can then be applied to models that predict SU trajectories to elucidate measurable changes in the brain that theoretically occur commensurate with behavioral change related to pathways for initiation and subsequent SU.

Illuminating interactions between neurobiological and social risk factors for, and effects of, SU on the development of neurocognitive skills has implications for designing interventions aimed at socio-cognitive-emotional regulatory processes, especially since such impairments can undermine intervention efforts (Riggs et al., 2006). The malleability of these neurocognitive processes may translate to protection against adverse outcomes, such as substance abuse and eventual dependence, in response to appropriately targeted interventions (Fishbein et al., 2016b). There is also potential for tracking change in neural substrates (e.g., task-related activation) in order to determine intervention effectiveness. Importantly, however, once task performance has been reliably linked to the recruitment of neural substrates relevant to SU pathways, the expensive imaging component can be discarded and replaced by substantially cheaper to administer tasks, which have been characterized as having functional neuroanatomical significance predictive of SU pathways.

A further advantage afforded by such research designs is the potential to dynamically explore moderators of neurodevelopmental and SU trajectories (e.g., sex, stress exposure and physiology, pubertal status, psychological traits, genetic markers, epigenetic variability). This exploration could provide information on subtypes of individuals who are at greatest risk for maladaptive outcomes, and thus can guide intervention delivery (i.e., which groups or subtypes should be targeted for intervention). In effect, findings that support the expectation that level of neurodevelopment will predict SU pathways and in turn, be influenced by subsequent use will suggest that early, targeted interventions that significantly and perhaps enduringly alter the developmental trajectory at critical time will lead toward more adaptive outcomes (i.e., no use).

Although there is great potential in the implementation of prospective, longitudinal neuroimaging studies of SU trajectories in adolescence, the inclusion of neuroimaging techniques also poses a new set of issues and challenges for prospective longitudinal study design. In this manuscript we describe the methodology that we adopted in our Adolescent Development Study (ADS), which was one of the first to adopt this novel application of tried and tested neuroimaging and developmental methodologies to questions of the precursors and consequences of adolescent SU. We focus on the key design aspects of our study and outline which aspects of our approach worked well, as well as critical challenges and pitfalls that we have faced. We also describe our “lessons learned” and how these might be relevant for future such endeavors.

## Adolescent Development Study

**Figure 1:**
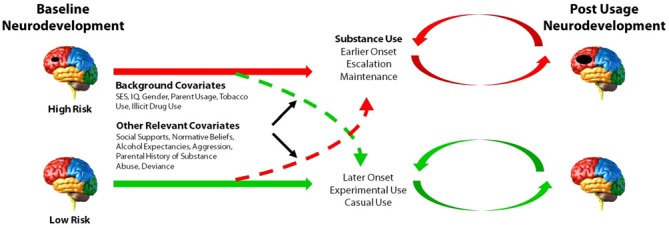
**Model of the main aims and predictions of the Adolescent Development Study (ADS).** Level of neurocognitive functioning, neuroanatomical development, as well as patterns of neural activation and connectivity at baseline are hypothesized to predict initiation and patterns of substance use (SU). Initiation and escalation of eventual use will, in turn, further aggravate neurodevelopmental outcomes over time relative to youth who do not use or who use later or in lesser amounts. Environmental and genetic factors may contribute to or dampen propensity to initiate and/or escalate SU.

The ongoing ADS (R01AA019983) was originally designed to identify neurodevelopmental “liability” factors that predate alcohol use and predict use initiation and escalation. We subsequently expanded our focus to all drugs of abuse. The model underlying this study postulates that baseline differences in neurodevelopment, in combination with a range of covariates (e.g., socioeconomic status (SES), gender, IQ, parental SU, genetic factors), will predict initiation and patterns of SU. Moreover, our conceptual model predicts that SU initiation and escalation will, in turn, aggravate neurodevelopmental outcomes over time in those who use illicit substances compared to those who do not use or who use later or in lesser amounts (Figure 1). The study has involved tracking neurodevelopmental trajectories in an initially SU naïve baseline sample (i.e., *N* = 135, 11–13 year olds) at three developmental time periods that are 18-months apart (i.e., baseline/Wave 1, Wave 2, and Wave 3). Level of neurodevelopment was reflected in measures of complex cognitive and regulatory functions (i.e., ECF and emotion regulation), and their neural substrates (e.g., brain morphometry and patterns of blood oxygen level dependent (BOLD) activation and connectivity in specific regions of interest) measured using structural and fMRI. Furthermore, ADS has tracked the impact of SU (initiation, quantity and frequency) on the trajectory of neurodevelopment. To-date we have tracked development from baseline to the first 18-month follow-up (i.e., Wave 2) and have just begun collecting the third wave of data. In the first 3 years of this 5 year effort, many challenges have been encountered, particularly regarding: (1) optimizing sampling to ensure sufficient numbers of eventual substance users (i.e., “high-risk” individuals); (2) recruitment of these often hard-to-reach populations; and (3) retention, given the duration between testing sessions, the lengthy, at times uncomfortable, test sessions, and the need for participants to travel to imaging facilities. These vagaries are common in longitudinal neuroimaging studies; thus, lessons learned will be imparted in this article.

The goal of ADS is to test the accuracy of our model in a community sample of alcohol- and drug-naïve youth. According to national surveys in community samples, about 12% of 15 year olds will report having been drunk and about 4% will have tried marijuana (Johnston et al., 2014); however, testing the proposed model requires a higher incidence of drug and alcohol use in our sample. Thus, we found it necessary to use an oversampling technique in order to ensure adequate numbers of “at-risk” participants who will initiate and accelerate drinking during the study. To this end, we prescreened potential participants and oversampled at-risk youth based on predictive traits (e.g., aggressiveness and other conduct problems, parental history of SUD). When effective, this strategy ensures that a sufficient number of youth will initiate and escalate use during the course of a 5-year longitudinal study, thereby capturing the full spectrum of adolescent SU patterns. Consequently, power is increased and thus the feasibility of using fMRI is ensured.

### Design

The prospective longitudinal design described herein enables both within- and between-subject comparisons to: (a) distinguish SU pathways in terms of pre-use cognitive function, neural activation patterns, and connectivity and subsequent neurodevelopmental trajectories; and (b) increase confidence regarding neurodevelopmental delays or deficits that can be attributed to SU. Three assessments spaced 18-months apart were planned with an initial projected sample of about 200 youth and their primary caregivers. A targeted sampling procedure with overlapping age groups (between 11 and 13 years old at baseline) in each wave of data collection was employed. This age range ensures inclusion of youth who are non-substance using at baseline and are entering critical developmental years during which initiation and escalation of SU are common and importantly, represents the age range at which SU is most likely to affect brain maturation. Although this approach has potential to underrepresent children at very high risk for SUD whose age of onset may be even earlier, inclusion of younger participants may impede the ability to follow children into an age range known for initiation and escalation in a 5-year study. However, expanding the age range introduces a large degree of developmental variation for which adjustments may be difficult. Recognizing these issues, an age range of 11–13 was determined to be an optimal compromise.

### Sample Selection and Recruitment

Households in the Washington, DC Maryland and Northern Virginia region were targeted on the basis of rates of SU, neighborhood crime, and income, while noting that even a “lower risk” county in the catchment area has adolescent alcohol use rates that increase substantially with age (see Table 1).

**Table 1:** **Alcohol and marijuana use prevalence (i.e., % of youth reporting lifetime, past-year, and past 30-day use) in the target population**.

Montgomery county adolescent survey: 2007	6th grade (11 years old)	8th grade (13 years old)	10th grade (15 years old)
**% of youth reporting lifetime use**
beer/wine	5.4	16.1	36.5
liquor	2.1	12.6	34.3
marijuana	1.1	5.1	17.8
**% of youth reporting past-year use**
beer/wine	4.0	13.4	32.7
liquor	1.3	11.4	30.1
marijuana	1.1	5.1	15.4
**% of youth reporting past-30-day use**
beer/wine	2.7	8.4	18.4
liquor	1.0	6.0	16.9
marijuana	1.1	3.3	9.6
**% of youth reporting 5+**	0.9	4.7	15.3
**drinks on same occasion in past 30 days (State-wide)**

*Adapted from the 2007 Maryland Adolescent Survey conducted on 2748 adolescents in Montgomery County Public Schools (http://marylandpublicschools.org/MSDE/newsroom/special_reports/adolescent_survey.htm. Accessed on 4/21/16)*.

A call center that supports survey research nationally was used to contact candidate families and to direct caregivers to complete a web-based risk screener to identify pre-adolescents at high risk. The Marketing Systems Group (MSG) was used to purchase listings (i.e., InfoUSA and Experian) of 40,000 current household addresses with updated telephone information, which was parsed with respect to geographic proximity to the Center for Functional and Molecular Imaging (CFMI) at Georgetown University (Washington, DC, USA) and predetermined to have a high probability of children 11–13 years old as well as an appropriate mix of socioeconomic characteristics. Using an established protocol to describe the study, its purposes and procedures, a call center contracted by RTI International (Dr. Fishbein’s primary institute at the time of study initiation) mailed introduction letters to 36,646 households followed by phone calls (Iannacchione et al., 2008). The call center staff determined initial eligibility and interest in about 2% of this sample. If a household was deemed eligible (i.e., a child living in the home and falling within the appropriate age range), they were asked to complete a web-screener to obtain additional information and determine eligibility at a more refined level (see web-based screening protocol below). Combined with other recruitment sources (e.g., Craigslist, in-person), we identified a total of 971 families that were identified as potentially eligible cases for the web survey. Additional strategies described below supplemented these listings based on successful efforts in large-scale longitudinal studies of high-risk adolescents (Trotman et al., 2014; Ridenour et al., 2015).

Web-based screening, the second phase of eligibility screening, was designed to further determine eligibility and oversample youth at high risk for SU initiation and escalation. The web-based risk screener is a brief 10-item survey, called the Drug Use Screening Inventory (DUSI-R) Quick Screen (DQS). It has been shown to be highly predictive (>73% in our baseline age range) of future SUD by the age of 22 (Kirisci et al., 2013a). A cut-off score of 5 on this survey is indicative of an increased risk of SU and provided the basis for oversampling of “high-risk” children, so as to ensure sufficient numbers who would initiate substance use by the 3rd wave of data collection.

Consenting parents/legal guardians were given a web link and confidential logon ID to respond to questions that identify age-eligible youth in the household at relatively high risk for substance misuse and other questions regarding eligibility. This web method is more likely to produce accurate responses to sensitive questions than telephone, computerized or interactive voice recognition interviews since respondents do not have to report to an interviewer and can self-administer the screener in privacy (Griffiths, 2010). Comprehension is also improved through visual presentation. Parents/legal guardians without access to the internet were asked to use their library or school, although ~70% of inner city minority residents now have access to the internet via web-enabled cell phones (US Census, 2010)^1^. For households that completed the telephone screener but indicated that they were unable to complete a web-based screener due to lack of reliable access to the Internet, the option was given to complete the screener by either mail or telephone interview. Individuals slow to respond to the web screener received reminder prompts via the method of their choice (either by phone, text, e-mail, or mail) to enhance participation. Caregivers received a check for $20 for completing the screener.

Exclusionary criteria, determined during screening, included caregiver reports that their child was severely emotionally disturbed or cognitively impaired, exposed to alcohol or illicit drugs prenatally, left-handed (i.e., a standard exclusion for fMRI studies due to lateralization of language function differences) as determined by the Edinburgh Handedness Inventory (Oldfield, 1971), a sibling of a current participant (i.e., to avoid complications due to clustering of environmental and genetic factors from within the home), and/or had a medical history that made it unsafe to participate in MRI.

Of the 971 cases identified by the Call Center and other sources, 26% were identified as eligible, with the web survey producing a much higher overall rate of about 50%. This strategy left us with 254 families that were eligible to be contacted for the telephone screener of both the parent and adolescent. Of those cases, 47% completed and were eligible, for a total of 115 families. Of those 115 families, 100 successfully completed Wave 1.

To attain a larger sample size, supplementary recruitment measures were taken. Flyers were posted and distributed in key areas where there is a known high concentration of either caregivers or children in the appropriate age range, such as stores, bus stations, unemployment agencies, clinics, courthouses, and newspapers. We also advertised on Facebook, Craig’s List and other social media sites, and sent flyers to pediatrician offices, community centers, and schools. Children were asked to bring the flyer to their caregiver, and instructed not to contact the study team or call center themselves. Flyers and advertisements instructed interested caregivers to contact the RTI call center to be screened for eligibility for further screening and enrollment in the study. Later in the process, contact was made directly with study staff rather than the call center, and we used the Georgetown Volunteer Research Program participant pool as a resource for recruitment. We conducted presentations at various locations including a health fair, a charter school expo, and various organizations that serve families in the DC region. In addition, we distributed our approved study flyers outside of malls and other public areas (e.g., libraries, stores, etc.). Our selective sampling for individual high-risk children was dropped at this point given the slow pace of recruitment, and instead we focused on specific locations within the catchment area considered to be at high risk. However, we retained other critical inclusion criteria, such as age, handedness, and substance use naiveté. RAs used a script for in-person recruitment in public areas, providing a flyer, answering questions about the study, and ascertaining inclusion criteria. If present, parents/caregivers were then offered a chance to complete the web-screener on the spot using an encrypted laptop in a private location, and were given a $20 cash incentive for completing the onsite assessment. If privacy could not be assured, the screener was not conducted and contact information alone was exchanged. Neither personally identifying information nor answers to the web screener were stored on the laptop. Children whose parents were not with them were handed a flyer with contact information to take home and discuss with their parents.

At this juncture, we had not yet achieved our recruitment goal; thus, we hired two professional recruiters to: (1) conduct a door-knocking technique in *a priori* defined high-risk neighborhoods; and (2) approach people in public areas, distribute flyers and verbally introduce and describe the study. A specific, non-random sample of household clusters in the DC area (i.e., households in regions where there were known high rates of those factors that contribute to high-risk designation) was identified for in-person recruitment. The sampling technique targeted areas at high-risk for substance use/abuse by identifying segments made up of census tracts within 20 miles of Georgetown where at least 60% of the population is 200% below the poverty level according to the most recent American Community Survey 5-year data. Individuals living within such neighborhoods are believed to be at increased risk for SU due to a myriad of detrimental socio-environmental factors, such as high rates of crime and high levels of drug use and trafficking. Segments with large college student populations were excluded as unlikely to be eligible. Within these segments, we randomly selected city-style mailing addresses from the U.S. Postal Service Computerized Delivery Sequence file and used a sampling rate not to exceed 1/3 within each segment.

Headway (a national job recruiter company) was contracted for experienced staff (1 English and 1 bilingual English/Spanish speaker) that had completed the Headway Fundamentals of Field Interviewing online course. Recruiters approached households to determine eligibility (i.e., an appropriately aged child living in the household) and conduct the web-screener on a secure and encrypted laptop. Headway staff have ample training and experience in recruiting difficult-to-reach populations and received further training on the purposes and procedures of the study, the focal population and geographic areas, approach, identifying the appropriate respondent, and methods to handle special circumstances. In particular, we implemented a protocol from previous studies that provides instructions about sensitivity to different cultural lifestyles and what to do in the case of witnessing violence, child abuse, partying, illicit drug use, and other challenging situations. They were also trained in ways to ensure privacy in the household and to protect confidentiality; similar to procedures we invoked in this study with our existing RAs. For completing the web-screener within the home, they asked respondents to move into a private location where they could not be heard, or to step outside for a few minutes. This strategy was effective, but was exorbitantly costly, and thus we were forced to terminate this approach in wave 1 before our target *N* of 200 could be recruited. Upon termination of this approach a total of *N* = 135 participants had been enrolled in and completed the first visit of wave 1.

Supported by a supplementary grant awarded in the third year of the study, an additional wave of recruitment is now underway to bolster our final *N*. The latest recruitment strategy is being conducted with the aid of two community organizations that service the most impoverished parts of DC: the DC Promise Neighborhood Initiative (DCPNI), whose mission is to end intergenerational poverty, and The Center of Excellence for Health Disparities (CEHD), whose mission is to eliminate health disparities in the District of Columbia by building bridges between researchers, policy-makers and the community. These organizations serve regions that have the highest rates of crime near their public schools in DC (District of Columbia Metropolitan Police Statistics Report, June, 2013)^2^ and have the highest reported rates of SUD across the entire DC Metropolitan Region (National Survey on Drug Use and Health 2008–2012)^3^. We have employed community-based recruiters identified by these two organizations who have prior experience recruiting for research studies. The community-based recruiters have been trained on the details of the study and the basic inclusion criteria: age-range and handedness. As with the previous recruitment approach, interested caregivers are given a flyer with information and a contact number at Georgetown University and are asked to contact the study team for eligibility screening and study scheduling.

**Figure 2:**
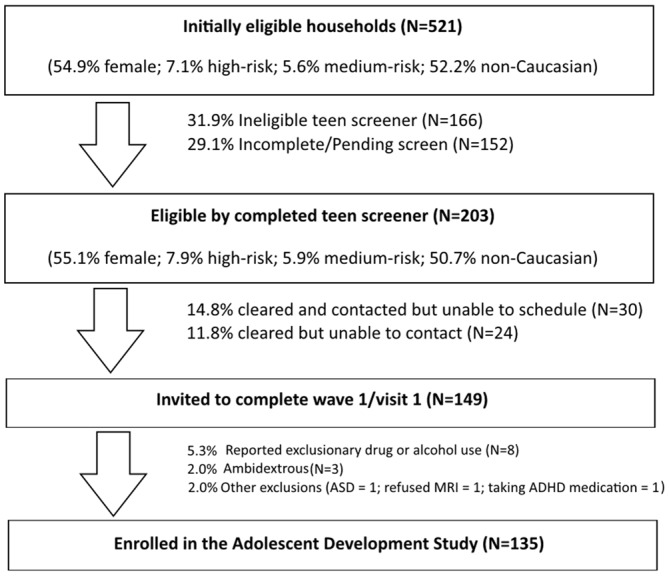
**Sample recruitment, screening, and interview outcomes**.

Figure 2 depicts the phased approach to recruitment collapsed across all strategies used (call center, community outreach, and targeted neighborhood recruitment, prior to a supplementary grant).

Of the families invited to complete the web-based SU screener, our goal was to identify a sample of SU naïve adolescents aged 11–13 years of whom 30% were at high risk for SU and 70% had lower risk levels (i.e., low and medium). Our estimates also factored in expectations for higher refusal and attrition rates in the high-risk group and differential completion rates for the baseline visit between risk-defined cohorts. It is important to note that although group assignment was useful for sampling purposes and has been applied to several analyses of baseline data, it was not the basis for primary analyses. Rather our analysis plans focus on actual SU and not risk *per se*. The use of risk categories in defining recruitment aims was simply to help ensure that a sufficient number of SU naïve adolescents would progress to use within the study timeframe.

Separate telephone calls with the parent and child were conducted for final screening. Included in the final screening were questions relating to routine medications the child was taking. Some medications classes were exclusionary criteria for this MRI study based on the evidence that they may have specific effects on blood flow, which could potentially interfere with the BOLD signal. The medication screener that we used in the survey had prompts to flag respondents who reported use of prescribed stimulant, allergy or other medications that may alter blood flow. The screener also queried medical and psychiatric disorders for which exclusionary medications may have been used. Those reporting medication use that did not meet those specific criteria or that are not known to be unrelated to brain blood flow were reviewed on a case-by-case basis to determine whether medication or related medical histories were exclusionary. Parents of children who were taking stimulants for ADHD, antihistamines for allergies or a cold, or other medications that potentially alter blood flow were asked if they routinely take short (e.g., 1–2 day) medication “holidays”. If so, the child was scheduled for a time during their regularly scheduled break in their treatment regimen (e.g., weekends or winter). Children taking these medications who did not take routine physician-recommended breaks were excluded at Wave 1/baseline.

In the final step of the phone screener, the child was asked to go to a room where he/she could speak in private to the interviewer. At this point in the interview the child was asked whether they had started using any alcohol or drugs, whether they had any body piercings, and questions to determine if they had ever suffered a concussion. At the end of the telephone surveys, and after it was confirmed that the child and their parent were interested in participation, parents were scheduled to bring the child in for the first visit.

## Study Procedures

The Georgetown University Institutional Review Board approved the study procedures and all youth and legal guardians gave informed assent and consent, respectively, prior to in-study participation. All assessments and interviews were conducted at CFMI for each youth and at least one primary legal guardian was required to accompany their child. Computer-Assisted Personal Interviewing (CAPI) was used to obtain background data and other non-sensitive information, while a Computer-Assisted Self Interview (CASI), more likely to elicit honest answers, was used to address sensitive questions regarding, for example, SU and maltreatment.

### Youth Interview

Prior to testing, youth were asked to refrain from ingesting caffeine-containing substances for at least 2 h. Although youth who reported using drugs or alcohol during the telephone screening were excluded prior to wave 1, questions related to SU were asked again at the baseline/wave 1 study visit in order to ensure a truly “clean” baseline (Note: this repetitive screening resulted in the exclusion of *N* = 8 scheduled Wave 1 participants). Additionally, six adolescents who had been recruited and scheduled were subsequently excluded for reasons not noted during the phone screening procedure. This included ambidexterity (*N* = 3), diagnosis of autism (*N* = 1), inability to abstain from ADHD medications (*N* = 1), and MRI refusal (*N* = 1). In waves 2 and 3, youth who have begun smoking are being asked to refrain from using nicotine for 2 h prior to testing and those who are inebriated or intoxicated will be excused and rescheduled, though this has yet to present as an issue.

In subsequent waves of data collection, extensive interviewing regarding SU, which is sensitive to increasing levels of use and detection of use before testing, has been included. The extensive questions in these interviews were framed to measure age of onset, quantity, frequency, cumulative exposure and “composite severity” for the use of a range of substances. Alcohol and drug consumption estimates and the presence of alcohol use disorder (AUD) and SUD in participants are obtained from both the subject and parent via the alcohol and drug portion of the Semi-Structured Interview for the Genetics of Alcoholism (SSAGA adolescent version; Bucholz et al., 1994). For the purposes of the study, we refer to this assessment as the Tobacco, Alcohol and Drug (TAD) questionnaire. Individual questions are based on well-validated items of other instruments, such as the Diagnostic Interview Schedule (DIS; Helzer and Robins, 1988). The TAD records age of onset, amount, frequency and duration of use, tolerance and withdrawal, and problem behaviors, along with social, physical, and cognitive effects of SU. The range of substances included in the TAD CASI interview included: tobacco, alcohol, marijuana, cocaine, methamphetamine, ecstasy, opiates, salvia, inhalants, and illegally used prescription drugs, along with an open-ended “any other substances” set of questions. Assessment items pertain specifically to both the period since the initial visit (i.e., the last 18 months) and past 30 days. This allows us to account for different drug-taking patterns between individuals and distinguish those who consume the same total quantity in a typical week but who may vary in use frequency (e.g., using a large amount of alcohol once or twice per week [i.e., “binge” drinking] vs. smaller quantities five times per week).

In all waves of data collection, the youth interview included the Scale of Physical Development (Carskadon and Acebo, 1993), the full DUSI-R to evaluate behavior, lifestyle, stress, family, and other relevant constructs, a sleep habits survey (Wolfson and Carskadon, 1998), and the Behavioral Inhibitory System/Behavioral Activation System (BIS/BAS; Carver and White, 1994) to assess inhibitory and arousal tendencies.

Given that childhood trauma is one of the most influential experiences in the causal sequence under investigation in the ADS, in the second wave of data collection we also began to administer the Early Trauma Inventory (Bremner et al., 2000). This measure allows for the assessment of several forms of childhood adversity and symptoms of traumatic stress. The questions are worded to elicit retrospective experiences, thus, the timing of assessment is not as important as other measures that must be collected at baseline.

### Primary Legal Guardian Interview

The parent/legal guardian interview at Wave 1 included questions that were used to establish SES, demographics, education, occupation, background, and behavioral factors. Demographic data on the children were collected in the web screener to adjust selection algorithms and maintain a sample proportionate to ethnic groupings and gender in the catchment area. The visiting parent provided information on family history of extent of problems with alcohol and illicit drugs in first- and second-degree biological relatives (e.g., parents, grandparents, aunts, uncles and siblings of the child). When possible, we contacted the other parent to obtain direct responses to this measure with respect to their half of the family tree; otherwise a waiver of active consent of a second parent was used. In adoption cases, caregiver information was captured to assess environmental influences but information on extended family was not collected.

The general ECF of the youth was rated by parents using the *Behavior Rating Inventory of Executive Functions* (BRIEF; Gioia et al., 2000), which allows for external validation of laboratory measures by measuring sub-domains of ECF in real-world settings and situations. We also administered a parent-report version of the full DUSI-R on the participating child during each data collection wave.

### Youth Neurocognitive Assessment

ECF and emotional regulation were measured using seven non-MRI, noninvasive, and developmentally appropriate tasks (see Table 2), which were specially designed to test functions related to activation in PFC and interconnected regions (e.g., anterior cingulate and amygdala). These tasks tap specific domains previously associated with risk for and actual substance misuse and are conducive to repeated administrations to gauge developmental change in the context of SU. Adjustments to scores on performance measures were made for intelligence (measured using the developmentally appropriate Kaufman Brief Intelligence Test (KBIT; Kaufman and Kaufman, 1990)). Experimental measures included the Cambridge Neuropsychological Test Automated Battery (CANTAB)^4^ tests for Spatial Working Memory and the Stocking of Cambridge and two tasks—Facial Recognition and Temporal Discounting that were administered using E-Prime Software. Prior to completing the Temporal Discounting task, participants were informed that they would receive a small reward (<$10) based on a random selection of one of their choices (Olson et al., 2007).

**Table 2:** **Computerized tasks used outside of the scanner and their associated functions**.

Task	Function
KBIT	Intelligence
Trail making tests (TMT)	Cognitive flexibility
Facial recognition task	Emotion perception
Rey auditory verbal learning test	Auditory-verbal learning/working memory
Spatial working memory task	Working memory
Stockings of cambridge	Problem solving
Temporal discounting	Reward sensitivity

### MRI Measures

A range of functional and structural MRI scans were collected on adolescents to measure neurodevelopmental factors related to our primary hypotheses; i.e., *adolescents with functional and structural prefrontal deficits will have lower neurocognitive and emotion regulatory functioning and thus increased likelihood of misusing substances* (for details see Table 3). These measures will also be used to determine the subsequent consequences of SU on brain development in adolescents that initiate use relative to their baseline substance-naïve cortical development and function.

**Table 3:** **Summary of MRI scanning parameters**.

Scan type	TR (ms)	TE (ms)	TI (ms)	No. Slices/Slice thickness (mm)	Effective Resolution	FOV	Flip angle	Matrix	GRAPPA acceleration factor/No. phase encoding lines
T1 MPRAGE	1920	2.25	900	176/1	0.97 × 0.97 × 1 mm^3^	250 × 250	–	256 × 256	–
DTI (80-direction)	7500	87	–	55/2.5	2.5 mm^3^	240 × 240	–	96 × 96	2/30
fMRI (task-dependent)	2500	30	–	47/3	3.0 mm^3^	192 × 192	90°	64 × 64	2/24
rsfMRI	2280	30	–	44/3	3.0 mm^3^	192 × 192	90°	64 × 64	2/24

Baseline MRI scanning included a high-resolution T1 MPRAGE anatomical scan that will be used for the consideration of variability in brain morphometrics (e.g., VBM, CT). In addition, an 80-direction DTI scan was collected. fMRI included a resting-state fMRI (rsfMRI) scan, to assess network connectivity when the brain was not engaged in goal-directed functions, and three fMRI scans designed to assess degree of neuronal function related to: (1) impulsivity; (2) ECF; and (3) reward sensitivity. The rsfMRI scan was acquired using the same parameters as other fMRI scans except with four less slices to reduce the TR for improved temporal resolution. All of these measures will be repeated at waves 2 and 3.

### fMRI Paradigms

#### Go/NoGo Task

Neuronal activity related to impulsivity (e.g., in the right inferior frontal gyrus) was measured using the Go/NoGo task (Menon et al., 2001; Simmonds et al., 2008). This task used a block design with alternating blocks of Go/NoGo (45 s) and Fixation (12–16 s) each repeated five times. During the Go/NoGo blocks a series of 30 letters was presented for 200 ms each, followed by a 1300 ms fixation. Subjects were instructed to press the button in their right hand as quickly as possible for every letter except the letter “Q”. A total of 150 letters were presented in this design of which 27 were the target letter “Q”, thus providing a sufficient number of individual events to accurately model “impulsivity” (i.e., inability to inhibit response to a “Q”).

#### Wheel of Fortune Task

The Wheel of Fortune task provides a measure of functional response to variable reward and loss probabilities, reward/loss anticipation, and magnitude of rewards/losses (Ernst et al., 2004; Smith et al., 2009). Task-dependent activity on this measure is usually seen in brain regions that constitute reward networks (e.g., OFC, nucleus accumbens). On each trial the participant was presented with a pie chart (i.e., the wheel), which visually represented the odds of winning either a large or small amount of money. For example, some proportion of the wheel was colored pink and represented the odds of winning a large amount of money, while the remaining portion was colored blue and represented the odds of winning a smaller amount. In this paradigm, the smaller monetary value always has a higher probability of winning. Participants completed a total of 90 trials. Across trials, the odds were randomly varied between 10% vs. 90% (32–42 trials) and 30% vs. 70% (48–58 trials) wheel configurations. For the 10/90 wheels, the dollar amounts used were split evenly between $9/$1 and $18/$2. For the 30/70 trials, the dollar amounts used were split between $7/$3 and $21/$9. In each trial, the wheel and dollar amounts were presented for up to 3000 ms or until the subject made a choice. The choice was followed by a 3000 ms delay, and then a 3000 ms feedback. During the feedback, the subject was informed of whether they won with their last selection or not, and presented with their running total. To encourage participants to make a selection for each trial, they were told ahead of time that if they did not respond quickly enough (i.e., within 3000 ms), they would automatically lose the higher dollar amount shown in the current wheel. The task was presented as a slow event-related design with temporal jitter provided by a variable between-trial fixation (i.e., 2500–10,000 ms set based on a Poisson distribution). While the amounts won or lost were theoretical and the participant was explicitly told they would not actually win the money, they were encouraged to try to maximize their gains. In light of the number of trials needed to accurately model brain activity for this measure, the number of event types and the variable response time for the participants, this task can be relatively long, and can take up to 21 min to complete depending on how rapidly the subject responds. Therefore, the task was broken up into three separate runs; in-between runs, the subject received verbal encouragement to attempt to exceed their previous total winnings.

#### Emotional Counting Stroop Task

Finally, the Emotional Counting Stroop task provided a measure of attentional bias for alcohol related words (Whalen et al., 2006), which has been reported in alcohol-dependent adults (Lusher et al., 2004). An attentional bias for alcohol-related words has been reported in dependent and non-dependent alcohol users (Bauer and Cox, 1998), suggesting that associations with alcohol salient stimuli may form prior to dependence. Variation in the attentional bias effect has also been found for cocaine use stimuli among cocaine-dependent men and women, showing a significant correlation between individual attentional bias effect for cocaine stimuli and distributed activations in the frontal, occipitotemporal, parietal cingulate, and premotor cortex (Kilts et al., 2014). Here this task was presented as a block design with 20 trials per block alternating between neutral, negative, or alcohol-related words. On each 1500 ms trial, the same word was presented vertically on the screen 1–4 times. Subjects were instructed to quickly press the button corresponding to the number of times the word was listed. This was important since reaction time (RT) is used to measure the Stroop effect between the different categories of words.

### Genetic and Epigenetic Biomarkers

Analyses in various populations suggest that genetic factors contribute significantly to variance in SUD liability (e.g., 40–60% for alcohol dependence; Rietschel and Treutlein, 2013). Using a combination of candidate-gene and genome-wide association studies, a number of gene variants have been identified that are believed to be of relevance to risk for developing SUD via an impact on intermediate phenotypes (i.e., changes in brain structure and function, cognition, behavior and affect). These phenotypes, in turn, may contribute to the likelihood that an individual will experiment with alcohol and/or drugs and the probability of use escalating to abuse or dependence. There is also potential for abused substances to dysregulate neuronal function through epigenetic modifiers that contribute to positive and negative emotional sequelae of SU and/or dependence (Koob and Volkow, 2010; Moonat et al., 2013). Using saliva and blood samples acquired from participants at baseline and follow-up, we plan to consider how associations between use status and neurodevelopment may be influenced by a selection of genetic and epigenetic measurements. DNA from the saliva samples was extracted using a modification of a previously described method (Freeman et al., 2003). Taqman single nucleotide polymorphism (SNP) Genotyping Assays were performed using an allelic discrimination assay protocol (Life Technologies, Carlsbad, CA, USA). Variable number tandem repeats (VNTRs) were amplified using specific primers, including one primer that was fluorescent-labeled. This amplification captures the region of interest as a PCR product. After amplification VNTR fluorescent-labeled products were analyzed using the 3730XL DNA Analyzer (Life Technologies, Carlsbad, CA, USA). Genotypes were identified using Genotyper software v5.0 (Life Technologies, Carlsbad, CA, USA).

Epigenetic regulation of neural pathways that support critical processes may contribute to neurodevelopmental changes associated with risk for substance misuse in adolescence and the consequences of adolescent SU (e.g., progression to abuse and dependence later in life (Spear, 2011)). Furthermore, early life adversity (a factor in SUD risk) may impact methylation of genes in the brain and peripheral tissues; a process that predicts adverse phenotypic changes (McGowan and Szyf, 2010). Thus, epigenetic changes (e.g., DNA methylation markers) may prove useful in understanding the impact of environmental factors on SUD risk and the potential for substance misuse to alter methylation. Using blood samples that are currently being collected from participants during wave 3, we will conduct an epigenome-wide association analysis using the Illumina Infinum Human Methylation 450 Bead Chip. This procedure will allow us to ascertain epigenetic changes that are related to baseline risk status, subsequent SU, and environmental factors occurring before and after SU. Since DNA methylation is potentially modifiable, these markers are likely to be excellent biomarker candidates for prevention strategies and for the delineation of prevention efficacy.

## Current Status

**Figure 3:**
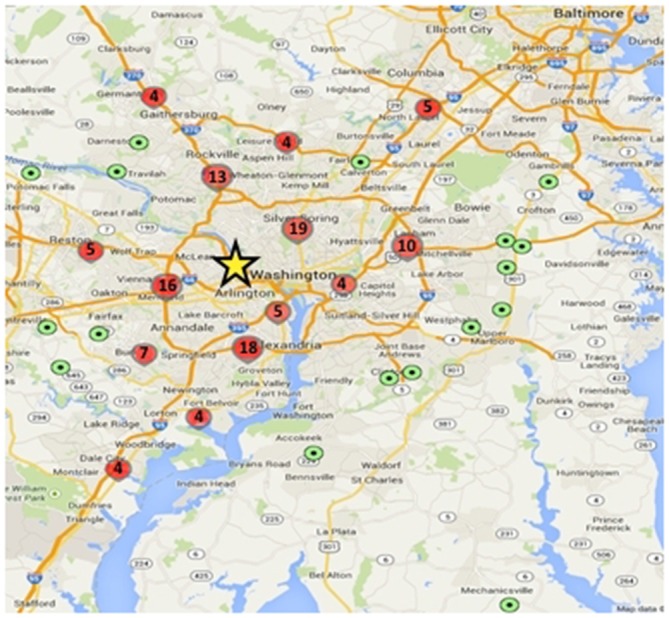
**Geographic distribution of study participants relative to testing center.** Green dots represent a single participant and red circles represent higher density locations (value within the circles denotes the number of participants within that circumscribed area); yellow star shows the approximate location of the Center for Functional and Molecular Imaging (CFMI), where the study takes place.

To date, 149 adolescents have been enrolled in the study, and 135 have completed Wave 1 data collection including MRI. Participants live throughout the greater Washington, DC region, with some traveling over 50 miles to participate, though most live within 15 miles of Georgetown University where the testing and scanning is conducted (Figure 3).

The baseline demographics for our sample are shown in Table 4. The sample is representative of the Washington, DC metro regional population, with 33% African American compared to 51% in the District, 30% in Maryland, and 20% in Virginia^5^. Although the median income is two brackets higher than the median for the region, the mean income bracket reported falls within the Census Bureau range.

**Table 4:** **Baseline demographics**.

	All	Females	Males	*p*
*N*	135	73	62	–
Age	12.7 (0.8)	12.6 (0.8)	12.7 (0.7)	0.886
Pubertal status	2.2 (0.70)	2.4 (0.8)	2.0 (0.5)	<0.001
Race and ethnicity				0.404
African American	45 (33%)	24 (32.9%)	21 (33.9%)	
Caucasian	70 (51.9%)	35 (47.9%)	35 (56.5%)	
Hispanic/Latino	9 (6.7%)	7 (9.6%)	2 (3.2%)	
Other	11 (8.1%)	7 (9.6%)	4 (6.5%)	
Socioeconomic status
Parent cumulative years education–mean (standard deviation)	16.2 (2.9)	15.9 (2.8)	16.6 (2.9)	0.173
Household income (*n* = 107*)
Mean	$50,000–$74,999	$50,000–$74,999	$50,000–$74,999	0.572
Median	$100,000–$149,000	$75,000–$99,999	$100,000–$149,999	
IQ (KBIT; *n* = 130**)	108.8 (15.3)	107.8 (13.4)	109.9 (17.3)	0.452
Alcohol risk distribution (from DUSI-R Quick Screen questions)				0.295
Low (%)	116 (85.9%)	62 (84.9%)	54 (87.1%)	
Medium (%)	8 (5.9%)	3 (4.1%)	5 (8.1%)	
High (%)	11 (8.1%)	8 (11.0%)	3 (4.8%)	

Notes: *Some individuals declined to report their income. **Some participants did not return for the visit during which the KBIT is administered.

Data collection for the first 18-month follow-up visit is completed, with ~87% (*N* = 118) having returned for their wave 2 testing. Data for 17 youth at Wave 2 were unavailable due to medical diagnosis (postural orthostatic tachychardia syndrome; *N* = 1), missing 18-month assessment window (*N* = 3), lost to follow up/unresponsive to calls (*N* = 3), relocation (*N* = 2), and unwillingness to continue participation (*N* = 8). Those unable to participate in MRI testing at wave 2 due to non-removable metal braces or exclusionary medication use completed all other aspects of the protocol including fMRI tasks in a mock scanner for behavioral data and will be contacted again for Wave 3. At baseline, 11 were identified as high-risk, 8 as medium-risk, and 116 as low-risk. Our extensive drug and alcohol use survey (i.e., TAD) shows that of the 118 participants for whom we have collected wave 2 data, we have confirmed drug/alcohol initiation of at least one substance in 12.7% (*N* = 15), 10 of whom are female. A further five participants have unconfirmed use at Wave 2 (i.e., they reported use of at least one substance on one SU measure, DUSI-R or TAD, but not on the other). We are currently awaiting follow-up to confirm use status when these participants attend for their Wave 3 visit. Among those who have confirmed SU initiation, only 33% (*N* = 5) were identified at baseline as high- or medium-risk for SUD, according to the DQS. However, the DQS is known specifically to predict SUD by 22 years of age and thus our high-risk cohort is still likely to initiate by Wave 3. Of those who have initiated, the most common substance used was alcohol (*N* = 11) although other drugs were also reported.

## Challenges, Pitfalls and Successes

The ADS has presented researchers experienced in longitudinal studies with many challenges that required continual monitoring, tweaking of design and methods, and consideration of alternative, more effective strategies. In effect, designing and programming the instrument battery, conducting the testing of participants once in the lab, and data analysis were the easy tasks since potential problems were anticipated and readily prevented or solved. Our sampling strategy was also well designed. The first tier oversampled in areas in the catchment area with high crime rates and low income but did not exclude lower risk regions. Although our goal was to obtain a representative demographic sample, neuroimaging studies tend to be small due to cost and burden, thus requiring sufficient numbers of high-risk pre-adolescents. Thus, the second tier of sampling focused exclusively on high-risk “pockets” (i.e., census tracts within 20 miles of Georgetown where at least 60% of the population is 200% below the poverty level according to the most recent American Community Survey 5-year data); a sampling strategy which proved effective.

Contacting and recruiting participants posed the greatest challenges. Based on experience, we correctly projected that only a small portion of our household listings would be both reachable and eligible. The success rate in terms of generating interest in proceeding on the phone and completing the web-screener was 39%. The contacting process was protracted, however, and we eventually exhausted the batches of listings we received prior to recruitment of sufficient numbers. Additional recruitment strategies were invoked, which were met with varying levels of success (see “Sample Selection and Recruitment” Section).

Another recruitment issue to consider is the relative success of individual approaches in sampling specific subtypes of participant, i.e., what proportion or total number of high-risk participants were recruited via each approach? This is an important consideration not only from the perspective of the number of high-risk participants, but also with regards to how many individuals eventually go on to initiate SU, and perhaps even the characteristics of SU. Although we failed to accurately monitor this in the ADS, we would highly recommend that other similar studies, which use multiple recruitment methods, carefully monitor this, and we certainly plan to do so in our own future endeavors.

To date, re-contacting initial participants for Wave 2 has been highly effective and although we did not meet our goal of 30% high-risk pre-adolescents, the numbers that have reported SU onset in Wave 2 (the first 18 month follow-up; i.e., 12.7%) came close to our projected estimates for Wave 2 use. Additionally, 9 of the 44 (20.5%) adolescents who have completed Wave 3 reported SU, including four participants who did not previously report SU at Wave 2. Importantly, using the wealth of knowledge gained from our initial recruitment efforts, we are now initiating a new strategy focused on recruiting 30 additional high-risk SU-naïve subjects (Note: high-risk status for this supplemental recruitment is defined in the same way as for participants from earlier recruitment efforts). While we will have only been able to collect two waves of data on these participants, it will be important to enrich our sample of adolescents known to be at high risk for SUDs. Community recruiters will identify potential subjects from the most impoverished parts of DC, giving participants’ contact information at GU. Only those subjects identified as high-risk for SU (i.e., according to previously outlined recruitment criteria) will be enrolled at this stage.

In addition to addressing recruitment challenges, we have attempted strategies that will ensure ongoing participation and limit attrition, such as structuring visits to limit participant fatigue while minimizing the number of visits and using strategies during the neuroimaging to ensure maximal cooperation. The amount of testing in each wave requires 6 h of the child’s time and 2 h of the parent’s time. To limit fatigue, testing is split into two 3-h visits, with the possible downside that the two visits per wave would lead to only partial completion of data collection. To mitigate the risk due to dropout between the two visits for each wave, the most important data are always collected in the first visit, i.e., the parent-provided information (demographics, family history of substance use, and parent assessment of child using the BRIEF), all of the neuroimaging, and the TAD. To maximize compliance during imaging, movement in the scanner is monitored, and participants are offered breaks and are allowed to watch a movie during the last 30 min of structural scanning. As a last resort, those subjects unwilling to return are asked for key information either via telephone or online.

An additional challenge has been neuroimaging quality; however, there are always expectations in neuroimaging studies that a reasonable percentage of scans will not be usable and those rates are factored into projections. In the current study, 83% of the baseline high-resolution structural scans have been checked for data quality using multiple raters and found to be usable for VBM and CT analyses based on independent visual inspection by three raters with an intraclass correlation of 0.94, comparable to other imaging studies.

## Discussion and Future Directions

The NIH is undertaking a national effort to commence a large-scale, prospective, longitudinal neuroimaging study for the next in 10 years—i.e., the Adolescent Brain Cognitive Development (ABCD) initiative—to isolate consequences from precursors of adolescent SU. Lessons learned from the current ADS effort suggest that it is critical for this and similar novel efforts to consider the looming challenges in terms of sampling strategy, recruitment, and retention for all subsequent efforts. Strategies for the ABCD effort are still under discussion and development, and longitudinal neuroimaging studies focusing on other outcomes are in conceptual stages; thus, a few recommendations follow.

First, ADS involved multiple institutions, similar to the strategy that is being undertaken for the ABCD initiative although on a much smaller scale. In a larger effort, it is deemed even more important that an innovative command and control framework implements best practices regarding team science. A scientifically collegial environment should be fostered that is organized to optimize distribution of scientific effort, maximize productivity, increase training opportunities, and consolidate investigator “buy-in” and commitment. Rather than a hierarchical structure, we recommend two-way communication across levels of authority, distribution of scientific effort, identification with the “we” in a horizontal organization as opposed to the “me” in a vertical organization, and agile responses to novel changes in circumstances. This structure facilitates both top-down and bottom-up communication via horizontal frameworks, as are often used in business “start-ups”. This horizontal structure also maximizes quick responses to challenges, which are likely to occur over the course of a longitudinal project, and facilitates the harvesting of high quality, actionable data for the broad scientific community and publications from its participating scientists. Rapid development and implementation of protocols is thus supported; additionally, there can be a facile shifting of resources from recruitment to retention of subjects, and investigator engagement to generate shared knowledge.

We also recommend a multi-stage school-based sampling plan nested within classrooms, schools, school districts and regional sites. This strategy may reduce labor costs and time associated with the techniques resorted to in ADS due to difficulties in recruiting overall, and in particular, in the targeting of high-risk individuals. A large probability sample receiving an intensive, empirically guided neurocognitive assessment significantly increases statistical power and generalizability. An important consideration (discussed later) is that community-based studies have the advantage of being able to collect data year-round and retain adolescents who may begin to attend school less frequently or eventually drop out. A strategy that partners with schools to assist in the recruitment strategy but avoids data collection in schools overcomes formidable logistical challenges of collecting data in schools, which threaten data richness, quality, and confidentiality.

Working with firms or institutes that have experience and a recognizable track record and reputation working with schools will aid in the initial sampling and recruitment (e.g., RTI International), although this can be a costly endeavor. An initial list of schools and publicly available information provides the pool from which a multiple level sample of school districts, schools, classrooms, and age-eligible children within the classroom can be drawn. Using standardized scripts, study staff members can conduct outbound telephone screenings of parents/legal guardians to identify those with an age-eligible child who are interested in participating. It is also critical that staff are trained on a common set of Frequently Asked Questions and are carefully selected and/or trained to be intimately familiar with the study’s purposes and procedures, and to relate to the respondents (e.g., education, etc.) with whom they will be interacting. As needed, we also recommend that studies consider the use of “replacement samples” to ensure that when individual subjects are excluded or withdraw after enrollment that subsequent recruitment targets individuals who are matched to these subjects on key criteria, thus maintaining the proportion of subjects from distinct cohorts “(e.g., high- vs. low-risk)”.

Based on prevalence of SU across any given catchment area compared to national data, the proportion of the target population coming from community sampling or high-risk sampling can be determined to meet two criteria: (i) potential selection bias to be below a specified rate and (ii) projected total number of substance users by a set age to exceed that of the national average on all major drug categories. As this is almost always guaranteed if the proportion of high-risk youth at each site is 50%, one can rely on community-sampling and high-risk sampling equally. To calibrate findings across studies, these enriched samples will need to be weighted back to their respective populations using the overlap of baseline risk status in the population and high-risk samples to form site-specific propensity scores (population vs. high-risk).

A selective recruitment oversampling strategy will ensure adequate variation on SU risk; we recommend a two-pronged approach. First, school-based recruitment can form the basis for generating a representative sample along with specific selection of children who are considered at particularly high-risk for SU on the basis of the school and neighborhood demographics or other individual-level criteria. Second, regional clinics can recruit additional children at high risk by virtue of childhood externalizing (ADHD, Conduct Disorder) and internalizing disorders (affective and anxiety disorders), a parent with a history of SUD or mental disorder, and other diagnostic indicators known to predict SU initiation and escalation. Alternative methods may also be employed, including using a combination of stratification of neighborhoods characterized by a high density of alcohol outlets and illicit drug use and venue sampling. Consenting parents can also be asked to complete a screener with a high level of accuracy in predicting SU; questions about parental SU can be included to enhance predictability.

To boost retention and follow-up, much depends upon engagement of parents/caretakers who may suffer from conditions that make retention in longitudinal studies difficult, such as SU, mental health issues, and homelessness. A standardized approach can facilitate implementation and monitoring across multiple sites. This pro-active model results in early detection of problems at individual sites and rapid problem resolution. The protocol includes the following steps: (1) collecting locator data for collaterals; (2) receiving information in a geospatial information system; (3) assigning each case to a follow-up case tracker; (4) verifying locator data; (5) outreach to unverified cases and weekly case review; (6) mailing thank-you cards to participants and collaterals; (7) scheduling follow-up appointments; (8) mailing post-enrollment flyers; (9) implementing returned-mail procedures; (10) calling participants before appointments to confirm date/location; (11) distributing a semi-annual newsletter and/or birthday cards; and (12) implementing a no-show protocol. Also, a coordinator can design and implement a “Brain Camp/Club” designed to balance assessment time with fun activities (“brain” games). Progress can then be monitored with daily reports and bi-monthly meetings by supervisory staff.

### Final Comments

Illuminating precursors for substance misuse and effects of SU on development of neurocognitive skills has implications for designing interventions aimed at socio-cognitive-emotional regulatory processes; a goal with great significance given that such impairments can undermine intervention efforts (Paschall and Fishbein, 2002; Aharonovich et al., 2003; Fishbein et al., 2005). Toward this goal, interventions can be designed and applied to catalyze morphological and functional neurodevelopment—specifically to optimize the development or enable acquisition of cognitive skills, emotional regulation, and behavioral self-control, thereby delaying initiation and long-term consequences of adolescent alcohol misuse. In addition, from a treatment standpoint, ameliorating neurological dysfunction or delays via cognitive and behavioral remediation, psychoeducation, speech and language therapy, and functional and integrative training would also appear to be useful approaches. In essence, the malleability of these neurocognitive processes (Hermann and Parente, 1996; Manchester et al., 1997; Fishbein, 2000; Riggs et al., 2006) may translate to protection against maladaptive outcomes (McGloin and Widom, 2001). We are hopeful that the findings from the ADS and similar studies in support of these hypotheses will suggest early, targeted interventions that may significantly and perhaps enduringly alter the developmental trajectory at a critical time point toward more adaptive outcomes.

## Author Contributions

DHF and JWVM were responsible for the conceptualization and design of the original study described in this manuscript. DHF, EJR, and JWVM and VLD contributed to the execution of the study. All authors were involved in the analysis and interpretation of the study-related information presented and all contributed to drafting and editing the important intellectual content of the manuscript. All of the authors give their approval of the version submitted and agree to be accountable for all aspects of the work.

## Funding

This research was supported by the following grants: NIH/NIAAA 1R01AA019983-01 and 3R01AA019983-02S1, NIH/NCATS 1KL2RR031974-01, and NIH/NICHD 2P30HD040677-11.

## Conflict of Interest Statement

The authors declare that the research was conducted in the absence of any commercial or financial relationships that could be construed as a potential conflict of interest. The reviewer DJM and handling Editor declared their shared affiliation, and the handling Editor states that the process nevertheless met the standards of a fair and objective review.

## Footnotes

^1^https://www.census.gov/2010census/

^2^http://mpdc.dc.gov/node/878852

^3^http://www.icpsr.umich.edu/icpsrweb/ICPSR/series/64

^4^http://www.cambridgecognition.com

^5^https://www.census.gov/2010census/
